# Exploring the neural basis for paternal protection: an investigation of the neural response to infants in danger

**DOI:** 10.1093/scan/nsz018

**Published:** 2019-03-07

**Authors:** Anna E van ‘t Veer, Sandra Thijssen, Jurriaan Witteman, Marinus H van IJzendoorn, Marian J Bakermans-Kranenburg

**Affiliations:** 1 Methodology and Statistics Unit, Institute of Psychology, Leiden University, Leiden, the Netherlands; 2 Leiden Institute for Brain and Cognition, Leiden University, Leiden, the Netherlands; 3 Department of Psychology, Education, and Child Studies, Erasmus University Rotterdam, Rotterdam, the Netherlands; 4 Leiden University Centre for Linguistics, Leiden University, Leiden, the Netherlands; 5 School of Clinical Medicine, University of Cambridge, Cambridge, UK; 6 Faculty of Behavioural and Movement Sciences, Vrije Universiteit Amsterdam, the Netherlands

**Keywords:** paternal protection, threat processing, protective behavior toward infant, fMRI, fathers

## Abstract

Perceiving potential threat to an infant and responding to it is crucial for offspring survival and parent–child bonding. Using a combination of functional magnetic resonance imaging and multi-informant reports, this longitudinal study explores the neural basis for paternal responses to threat to infants pre-natally (*N* = 21) and early post-natally (*n* = 17). Participants viewed videos showing an infant in danger and matched control videos, while instructed to imagine that the infant was their own or someone else’s. Effects were found for infant-threatening *vs* neutral situations in the amygdala (region-of-interest analyses) and in clusters spanning cortical and subcortical areas (whole-brain analyses). An interaction effect revealed increased activation for own (*vs* unknown) infants in threatening (*vs* neutral) situations in bilateral motor areas, possibly indicating preparation for action. Post-natal activation patterns were similar; however, in part of the superior frontal gyrus the distinction between threat to own and unknown infant faded. Fathers showing more protective behavior in daily life recruited part of the frontal pole more when confronted with threat to their own *vs* an unknown infant. This exploratory study is the first to describe neural mechanisms involved in paternal protection and provides a basis for future work on fathers’ protective parenting.

## Exploring the neural basis for paternal protection

Parental protection is indispensable for a child; parents provide shelter, ward off (outgroup) hostility and safeguard children from harm. Despite the obvious importance to child survival, parental protection has received little attention in the literature to date. Similar to many intuitive prosocial behaviors, this type of anticipating and responding to a child in danger is likely rooted in neural systems previously found to be implicated in other forms of parental care (cf. Preston, 2013). Like caregiving, differing strategies of protection of the infant have been suggested to lead to different developmental outcomes (George and Solomon, 2008; van IJzendoorn and Bakermans-Kranenburg, 2018). From an evolutionary perspective, protection by parents is—alongside its symbiotic relationship with a newborn’s innate bias to seek a responsive attachment figure—crucial for survival. In this study, we explore the neural basis for paternal protection by assessing neuronal responses of fathers to situations that threaten their (imagined) own or an unknown infant, both before and after the birth of their first child. In addition, we investigate the association of this neural signature with real-life protective behavior using a multi-informant approach.

As human infants are vulnerable for a relatively long time, it is likely that human parents have evolved adaptive responses to infant-threatening situations, such as the threat of an accident (Hahn-Holbrook *et al*., 2011). Despite its evolutionary importance, few studies investigate the mechanisms of parental protection (Bakermans-Kranenburg and van IJzendoorn, 2017). This is surprising, because the parent’s active engagement in protective behavior may be a valuable marker for parent–child bonding and later child development. Studies of non-human animals suggest the great advantage of a sense of security and protection during early life for the ability to develop social bonds later in life, which in turn may maximize reproductive success (Sachser, 2005). Likewise, in humans, close relationships signal that the world is a safe environment where one is protected and serve distress alleviation regulatory functions (Bowlby, 1973, 1988). Moreover, the importance of parental protection becomes evident when protection is absent. For instance, child maltreatment—arguably the opposite of protection—is associated with impaired cognitive and emotional functioning later in life (e.g. De Bellis *et al*., 2009; Pechtel and Pizzagalli, 2011). By studying the mechanisms for parental protection, researchers can begin to shed light on the network of possible neurobiological, behavioral, developmental and societal antecedents and consequences of this dimension of parental care.

Parental caregiving is rooted in subcortical-paralimbic structures involved in emotional processing, enabling parents to automatically detect and respond to survival-related cues, and structures involved in social understanding, such as the medial pre-frontal cortex, superior temporal sulcus (STS), frontopolar cortex and temporal poles, allowing parents to understand their infant’s needs (Frith and Frith, 2006; Atzil *et al*., 2011; Barrett and Fleming, 2010; Abraham *et al*., 2014; Kringelbach *et al*., 2016; Rilling and Mascaro, 2017). Although much of what is known about the parental brain comes from studies with mothers, recent studies suggest that fatherhood is associated with both structural and functional changes in the brain (for a review, see Feldman, 2015). For instance, Kim *et al*. (2014) found gray matter volume increases in first-time fathers in regions associated with reward, affiliation and processing of infant stimuli, such as the striatum, amygdala and hypothalamus. Furthermore, Abraham *et al*. (2014) found that the amount of time fathers spent caring for their child correlated with amygdala–STS connectivity. These findings suggest that fathers’ brains attune to their caregiving role and that similar brain regions may be involved when fathers perceive a threat to their infant.

Studies that have examined paternal responses to infant cues have outlined brain regions previously implicated in reflexive caregiving, emotion regulation, executive function and empathy (Swain *et al*., 2014; Swain, 2017). Differences in activation patterns have been found when fathers perceive their own *vs* someone else’s infant or *vs* adults, although research in this area is still in an early stage (Platek *et al*., 2004; Atzil *et al*., 2012; Kuo *et al*., 2012; Wittfoth-Schardt *et al*., 2012; Mascaro *et al*., 2013). Differences in the perception of infant faces modified to display more or less kinship cues are often explained by selection pressure for fathers to recognize and invest in their own kin (for a review, see DeBruine *et al*., 2016). In a similar vein, protection may, like other prosocial behaviors, be preferentially expressed toward kin (Geary, 2006; Alvergne *et al*., 2009; Arseneau *et al*., 2015; Decety *et al*., 2016). We therefore examined fathers’ neural responses to potential threats to their own *vs* an unknown infant. Additionally, because the amygdalae have frequently been implicated in regulating responses associated with social cognition, including salience detection, threat processing and familiarity (Davis and Whalen, 2001; Adolphs, 2010; Pessoa and Adolphs, 2010; Natu and O’Toole, 2011; Duvarci and Pare, 2014), we targeted this subcortical region as region of interest (ROI) to examine the paternal neural response to infants in danger.

Despite the multitude of research on the processing of threatening signals, the neural mechanisms associated with paternal processing of threat to infants have remained uncharted. In the present longitudinal study, we explore this domain both prior to and after the birth of the father’s first child, and importantly, we examine how interindividual differences in brain response relate to differences in everyday protective behavior. To this end, we examine, first, expectant fathers’ neural response to videos depicting infants in threatening situations compared to neutral situations, asking the fathers to imagine that the infant was either their own or someone else’s. Secondly, we investigate the relation between threat processing and everyday actions of protecting the unborn child (e.g. preventing the pregnant mother from having to lift heavy objects). Lastly, as exposure to a child has been associated with both neural and hormonal changes in men (Wynne-Edwards, 2001; Rilling, 2013; Feldman, 2015), we investigate whether processing threat to one’s own or an unknown infant changes once the father’s child has been born.

## Method

### Participants

Twenty-five first-time fathers-to-be participated. Sample size was determined by the ethics approval request. Participants were recruited online and through midwives. Pre-selection criteria were cohabiting with their pregnant partner (second half of pregnancy), fluency in Dutch and good health, without (using medication for) psychiatric, neuroendocrine or neurological disorders. Participants were screened to exclude claustrophobia, metal parts in the body, excessive smoking and/or alcohol use, recreational drug use within 6 months prior to participating and use of steroidal or any other interfering medications.

Due to technical issues (several videoblocks contained both neutral and threatening videos instead of one video type), data of four participants in the pre-natal session were unusable. In total, 21 fathers-to-be (*M*_age_ = 31.48, *SD*_age_ = 3.97, range 24–39 years) were included in the final analysis of the pre-natal session. The mean gestational age of the child was 25.58 weeks (*SD* = 4.58, range = 21–37 weeks). During the post-natal follow-up, data of 17 fathers were obtained, and infants were between 12 and 20 weeks old (*M*_age of infant_ = 15.85, *SD*_age of infant_ = 2.66). Participants were instructed to abstain from alcohol and excessive physical activity during the 24h before the start of each session and from caffeine on the day of the session. Both functional imaging sessions took place at similar times of day. Written informed consent was obtained according to the declaration of Helsinki, and subjects received financial rewards of €30 per visit and €10 if the participant and their partner completed online questionnaires at home.

The study protocol was approved by the ethics committee of the Institute for Education and Child Studies at Leiden University and by the Medical Ethics Committee of the Leiden University Medical Center. We report sample size determination, all data exclusions, all manipulations and all measures associated with the current paradigm. This study was part of a larger study in which—in order to investigate the role of hormones in paternal behavior—participants self-administered vasopressin (AVP) intranasally during another pre-natal laboratory visit (see Thijssen *et al*., 2018 for effects of AVP on the processing of infant cry sounds). For comparison with the post-natal visit, data from the pre-natal placebo visit were included in the current analyses.

### Procedure

Both pre-natal and post-natal laboratory sessions had identical procedures, starting with instructions about the experiment, self-administration of a placebo and a brief training of the task on a laptop, which familiarized participants with the task and the infant pictures that were used to facilitate imagination of the child’s identity (own and unknown). In order to create a depiction of the father’s own (unborn) child, a morphed picture was created from a photograph of the participant (see below). It was explained to the participant that this could resemble a future child of his. A resting-state scan and working memory functional magnetic resonance imaging (fMRI) paradigm (to be reported elsewhere) preceded the current task. In the week after the first visit, fathers-to-be and their pregnant partners independently completed an online questionnaire including the Paternal Protection Questionnaire (PPQ; see below).

**Fig. 1:**
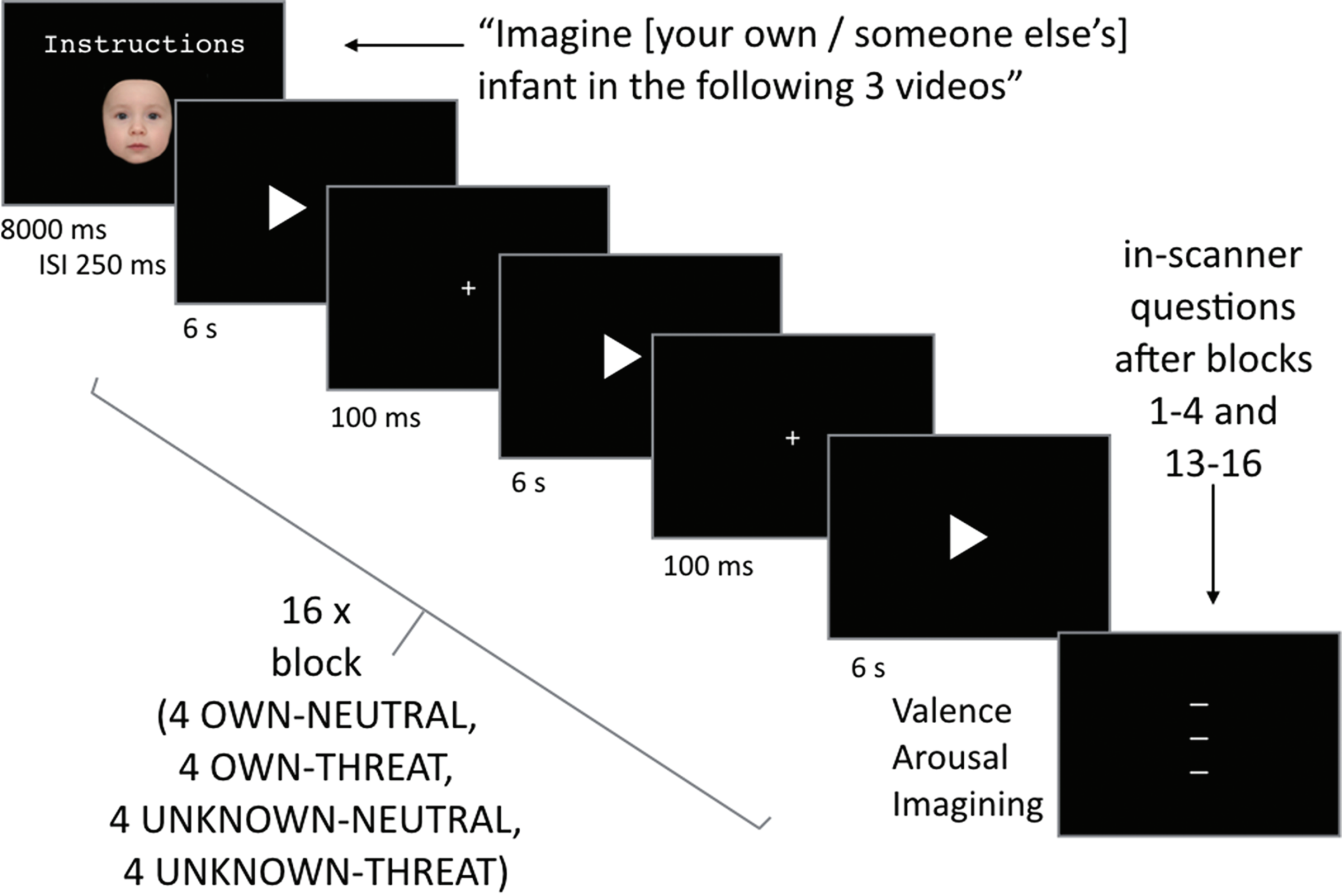
fMRI paradigm designed to assess neural processing of own (*vs* unknown) infant in threatening (*vs* neutral) situations. All stimuli were placed on a black background (in E-Prime 2.0) and were presented via a beamer that projected onto a screen placed in the back of the MRI bore, visible to participants via a mirror on the head coil.

### Measures

#### fMRI paradigm

To assess neural processing of threat to an infant, participants viewed threatening and neutral videos (threat factor) while imagining that either their own or someone else’s infant (familiarity factor) was shown in the videos (see Figure 1). This resulted in 4 conditions, each repeated 4 times, making 16 blocks overall. Each block consisted of three consecutive 6 s videos. Participants were randomly assigned to one of the four pre-programmed block orders, which ensured that each condition was present in each quarter of the task. Before a given block, to facilitate imagining own or unknown infant in the videos, participants saw a morphed picture of either their own infant or someone else’s infant accompanied by the instructions to imagine that this infant would be the infant in the subsequent videos. These pictures were made by combining 75% an average baby face (Hahn *et al*., 2015, 10 female and 10 male infant faces averaged) with 25% of either a picture of a male face unknown to the participants or a picture of the participant himself using the program Fantamorph (see Supplementary data).

After 8 s (and a brief stimulus interval of 250 ms), the instruction screen advanced to a block of three threatening (neutral) videos that each lasted 6 s (block length 18 s). Videos were semi randomly selected out of a pool of six threatening videos (i.e. hot tea is accidentally dropped on a baby, a baby stroller accidentally rolls into a river, an adult loses grip of a baby stroller that rolls off a bridge and crashes into a cyclist, a car seat with a baby is accidentally pushed and falls down stairs, a baby accidentally falls off a changing table while being changed and a car parks backwards and hits a baby in a car seat placed on parking lot) and six matched neutral videos (e.g. tea is placed on a table next to the baby, a baby stroller does not roll into the river, an adult on top of a bridge safely puts baby stroller on the brakes, a baby lies on the changing table while being changed and a car parks backwards a safe distance away from a baby in a car seat placed on sidewalk). These videos thus contrast situations in which protective action is called for, and situations in which it is not. The videos (available upon request) were filmed using a lifelike baby doll by a professional video production team. In order to ease the task of imagining own or unknown infant in the videos, care was taken to minimize depiction of the doll’s face or that of any actors. In order to further facilitate imagining the identity of the infant, two blocks with the same infant were presented consecutively.

After the first four blocks and after the last four blocks participants were asked to report their feelings of arousal [‘How calm or tense do you feel right now’, calm (0)–tense (100)] and valence [‘How positive or negative do you feel right now?’, negative (0)–positive (100)], and how well they succeeded in imagining their own or someone else’s infant in the preceding block of videos [very poorly (0)–very well (100)] on a visually presented scale by sliding to the right or left with right-hand button presses.

#### Paternal Protection Questionnaire

In order to measure the fathers’ daily protective behavior toward their unborn child, we developed the PPQ consisting of 20 items about the father’s behavior in the past week, e.g. “I paid attention that my partner did not eat something that could harm the baby”, “I helped my partner take rest” and “I let my partner lift heavy items” (reverse coded). An exploratory factor analysis was conducted, resulting in eight items loading > .4 that were retained (α = .82). Because protecting an unborn child is inherently related to protecting and taking care of the child’s mother, and because the father’s self-report may be biased, we also assessed the mother’s perspective on the father’s behavior with the same items adapted to mother’s perspective (α = .71) and used a multi-informant method, canceling out the weaknesses of one informant’s perspective with the strengths of the other’s (Kraemer *et al*., 2003). The father’s and mother’s perspectives were integrated by subjecting their responses on the eight items to a principal-components analysis, of which the first component reflected the report of both informants. Both informants contributed uniquely, as father-report and mother-report were less correlated (*r* = .41, *P* = .08) than the multi-informant measure was with the father’s (*r* = .93, *P* < .001) and the mother’s report (*r* = .68, *P* = .001). This multi-informant measure of paternal protection was subsequently used in analyses.

### fMRI data acquisition

Images were obtained on a 3T Philips Achieva MRI system (Philips Medical Systems, Best, the Netherlands) at the Leiden University Medical Center with a 32-channel SENSE (Sensitivity Encoding) head coil. A block design with 295 T2^*^-weighted whole-brain echo planar images, repetition time (TR) = 2200 ms was used for the functional scan, scan duration 11 min [echo time (TE) = 30 ms, flip angle = 80 °, voxel size = 2.75 × 2.75 × 3.025 mm with a 10% interslice gap, 38 transverse slices, field of view in mm = 220 × 220 × 115 (RL, AP, FH, respectively). The first five functional volumes were dummies to allow for steady-state tissue magnetization. For registration purposes, an anatomical 3D T1-weighted scan was obtained (TR = 9.7 ms, TE = 4.6 ms, flip angle = 8°, 140 transverse slices (voxel size .875 × .875 × 1.2 mm).

### Pre-processing and analysis of functional imaging data

Image processing was completed using FEAT (FMRI Expert Analysis Tool) Version 5.0.8, part of FSL (FMRIB’s Software Library, www.fmrib.ox.ac.uk/fsl). Registration of the functional data to the high-resolution structural image of each participant was carried out using the boundary-based registration algorithm (Greve and Fischl, 2009) and registration of the high-resolution structural image to standard space was carried out using FLIRT (Jenkinson and Smith, 2001; Jenkinson *et al*., 2002; linear registration was applied because non-linear registration did not perform well for some participants). The following pre-statistics processing was applied; motion correction using MCFLIRT (Jenkinson *et al*., 2002), non-brain removal using BET (Smith, 2002); spatial smoothing using a Gaussian kernel with a full-width-at-half-maximum of 5 mm; grand-mean intensity normalization of the entire 4D dataset by a single multiplicative factor; and a high-pass filter cutoff of 90 s (Gaussian-weighted least-squares straight line fitting, sigma = 45.0 s).

Time-series statistical analysis was carried out using FILM with local autocorrelation correction (Woolrich *et al.*, 2001), double-Gamma HRF convolution and temporal derivatives for the regressors of interest. Regressors were the onsets of blocks of three videos belonging to one of the four conditions in the 2 (threat *vs* neutral) × 2 (own *vs* unknown) design. Presentation of the pictures, in-scanner questions and movement [motion parameters and additional pre-computed motion outliers (fsl_motion_outliers; DVARS, http://fsl.fmrib.ox.ac.uk/fsl/fslwiki/FSLMotionOutliers)] were added as additional confound EVs.

### ROI analysis

Previous research suggests that especially the amygdalae are involved in salience and threat detection (for a review, see e.g. Duvarci and Pare, 2014). Furthermore, a reverse-inference map created with Neurosynth, an automated neuroimaging meta-analysis tool, shows the presence of the term ‘threat’ in article abstracts is predominantly associated with activation reported in the amygdala (http://neurosynth.org/analyses/terms/threat/, July 2018, 170 studies). Given our interest in processing of different threats, we ran the 2 (threat *vs* neutral) × 2 (own *vs* other) model on these subcortical regions. We structurally defined the right and left amygdalae using the Harvard-Oxford subcortical Structural Atlas and used these as a weighted mask image in Featquery (FSL, https://fsl.fmrib.ox.ac.uk/fsl). Mean *Z-*values averaged over all weighted voxels within bilateral amygdala were extracted for each subject and condition and were subsequently analyzed in SPSS with GLM Repeated Measures (data and syntax available on https://osf.io/z9r5t) and plotted in R.

### Group level whole-brain analysis

Group analysis was carried out using FLAME (FMRIB’s Local Analysis of Mixed Effects) stage 1 with automatic outlier detection (Beckmann *et al*., 2003; Woolrich *et al*., 2004). Z (Gaussianised T/F) statistic images were thresholded using clusters determined by *Z* > 2.3 and a (corrected) cluster significance threshold of *P* = .05 (Worsley, 2001).

Three different group level analyses were conducted. First, whole-brain analysis of the main effects of threat [i.e. threat (threat-own and threat-unknown) *vs* neutral (neutral-own and neutral-unknown)] and familiarity [i.e. own (threat-own and neutral-own) *vs* unknown (threat-unknown and neutral-unknown]) and their interaction were modeled [i.e. (threat-own and neutral-unknown) *vs* (threat-unknown and neutral-own)]. Secondly, this analysis was repeated with paternal protection as a covariate. Finally, we sought to document any changes of the task effects post-natally and performed a group-level repeated GLM to learn whether any regions show significant differences between the pre-natal and post-natal sessions in the 17 participants who completed both sessions. Significant clusters for interactions were followed up by extracting the mean *Z-*values with Featquery (FSL, https://fsl.fmrib.ox.ac.uk/fsl) and plotting the means in R to interpret the interaction. Group level maps are available on https://neurovault.org/collections/4122/. For a de-identified dataset with mean *Z-*values, multi-informant PPQ and in-scanner ratings, see https://osf.io/z9r5t.

## Results

### In-scanner ratings

We performed separate 2 (threat *vs* neutral) × 2 (own *vs* unknown infant) × 2 (time point in the task, beginning *vs* end) repeated measures ANOVA on participants’ in-scanner ratings of arousal, valence and how well they succeeded in imagining their own or someone else’s infant (for full details, see Supplementary data). As expected, participants reported to feel more tense and more negative after viewing threatening (*vs* neutral) situations, especially when their own infant (*vs* an unknown infant) was in danger (interaction threat and familiarity, *P*’s < .005, *η*_p_^2^ > .30). There were no significant differences on how well they did at imagining own or unknown infant between the four conditions, and means for this rating were above the midpoint, suggesting participants did not have trouble imagining the identity of the infant. Time point in the task did not have strong effects on participants’ ratings, although for threatening situations they felt more negative in the beginning of the task compared to the end. Importantly, these results were highly comparable between the pre-natal and post-natal session, meaning the in-scanner ratings for the different conditions were similar before and after birth of the baby (see Supplementary data).

### Whole-brain analysis

**Fig. 2:**
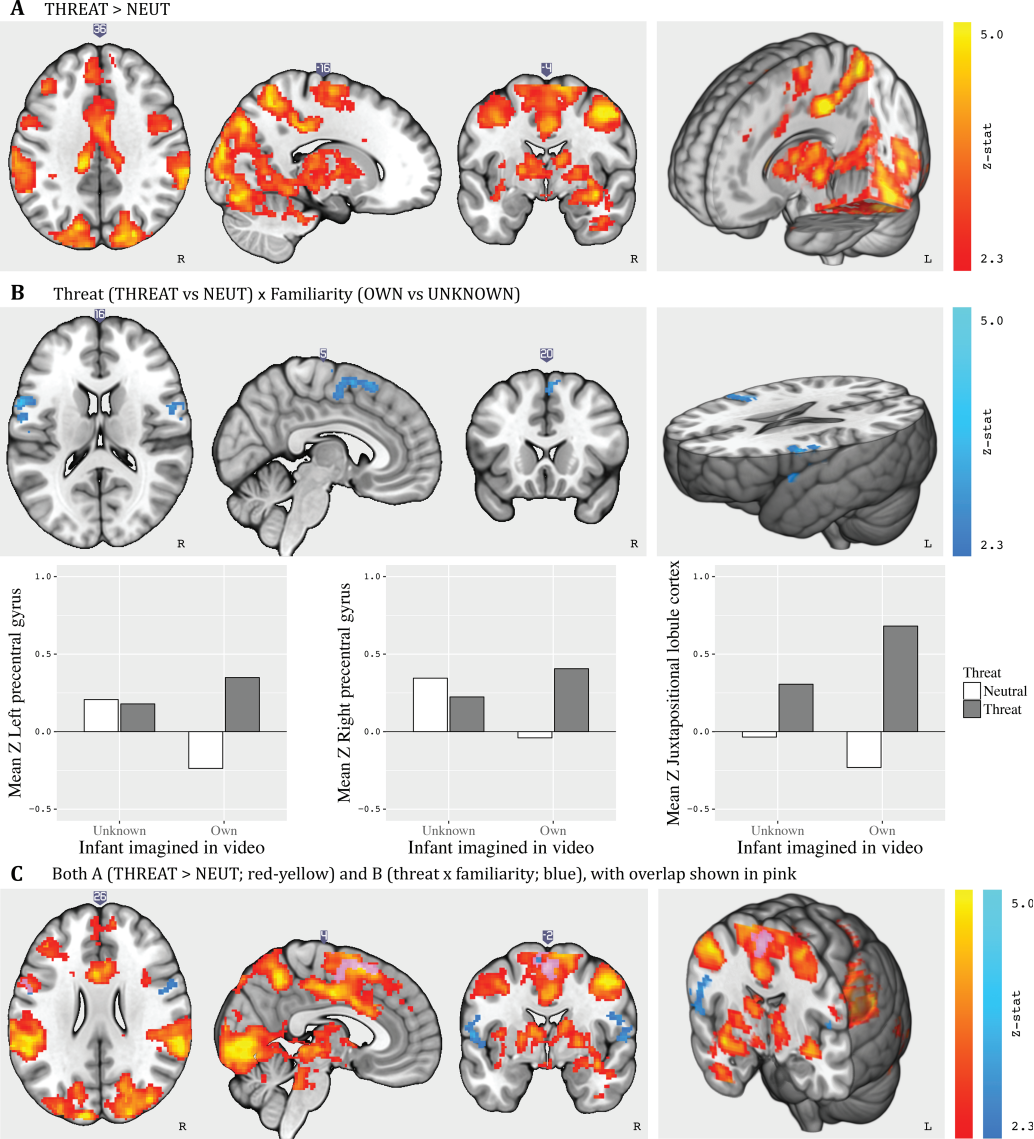
Activation for (A) main effect of threat, (B) interaction between threat and familiarity and mean *Z*-values for the interaction between threat and familiarity in the left pre-central gyrus, right pre-central gyrus and juxtapositional lobule cortex, (C) combination of A and B. Results (cluster-thresholded at 2.3, *P* < .05) are displayed on MNI-normalized template brain (i.e. mni152_2009bet.nii) using MRIcroGL software.

We first ran a group analysis on the pre-natal run to assess the two-sided contrasts of threat *vs* neutral situations, own *vs* unknown infant and their interaction. The main effect of threat *vs* neutral showed significant increased activation in response to threatening videos in three clusters (see Figure 2A, see next page and Supplementary Table 1). The first cluster included (bilaterally) the parietal operculum, the posterior cingulate cortex, the lingual gyrus, the occipital pole, the lateral occipital cortex and the juxtapositional lobule cortex [*P* < .001, formerly the supplementary motor area (SMA)/pre-SMA]. The second cluster included the left middle frontal gyrus (*P* = .003), and the third included the right lateral ventricle (*P* = .045; possibly a task-correlated artefact). As can be seen in Figure 2A, next to the above-reported local maxima areas, the effect of threat spans a variety of brain areas, including the insula, superior frontal gyrus (SFG), pre-central gyrus, superior parietal lobule and anterior cingulate gyrus. Next to this, activation was also found in subcortical areas including the midbrain and striatum, areas previously associated with parental motivation (Kim *et al*., 2014). The main effect of familiarity (own *vs* unknown infant) did not show significant activation.

Additionally, threat and familiarity interacted in three clusters, with activation being larger for own (*vs* unknown) infant in a threatening (*vs* neutral) situation. The first cluster predominantly consisted of the left pre-central gyrus and left central opercular cortex (*P* = .006). The second cluster consisted (bilaterally) of the juxtapositional lobule cortex and SFG (*P* = .010). The third cluster included the right pre-central gyrus and central opercular cortex (*P* = .041; Figure 2B and C and Supplementary Table 2). The main and interaction effect did not differ as a function of session order (i.e. whether the placebo session of a particular participant was his first or second time completing the task), see Supplementary data.

### ROI analysis

**Fig. 3:**
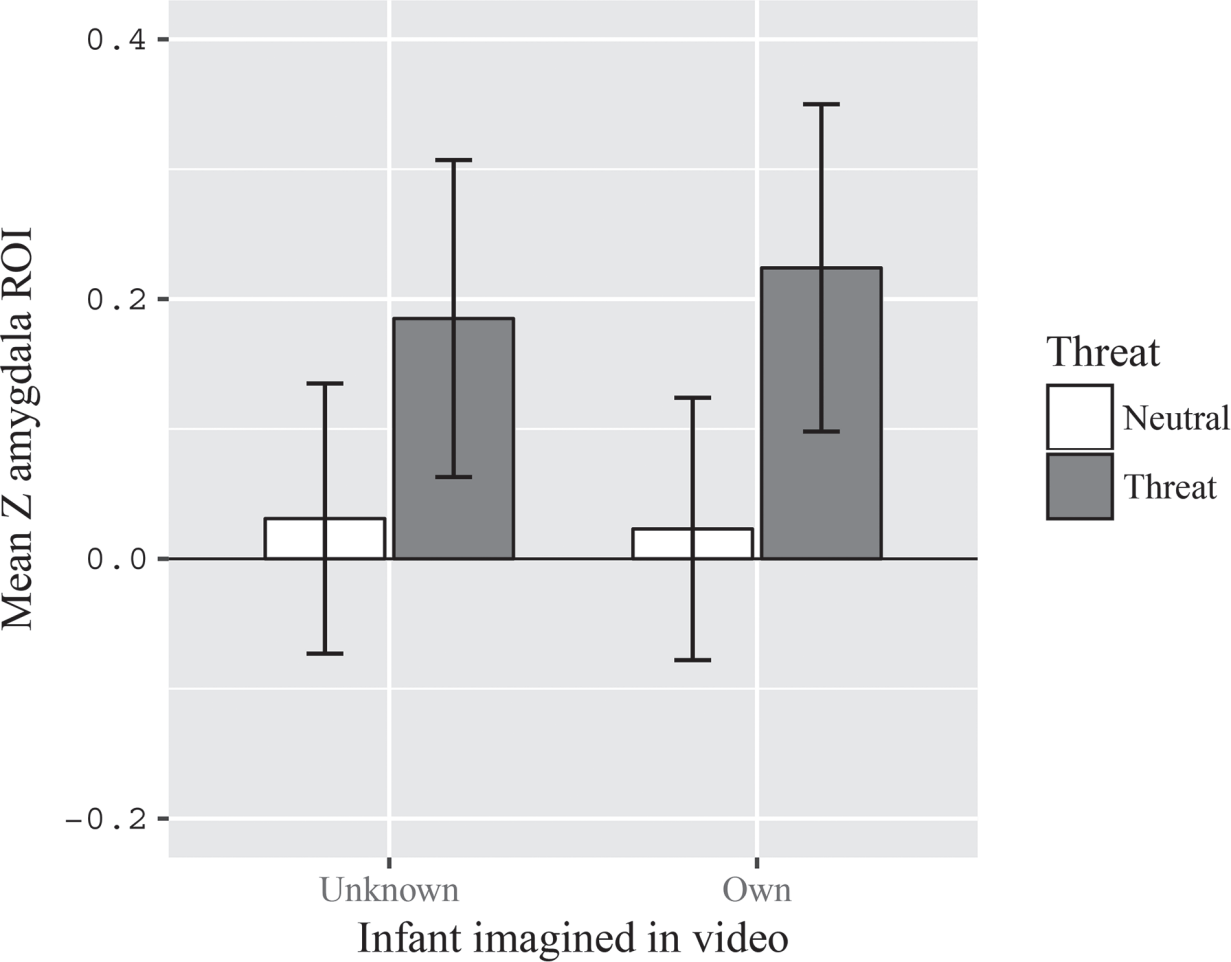
Amygdala activation as a function of threat (threat *vs* neutral) by familiarity (own *vs* unknown infant). Activation was higher for threatening situations compared to neutral situations. Error bars represent standard errors of the mean.

ROI analysis consisted of running the 2 (threat *vs* neutral) × 2 (own *vs* unknown infant) model on the mean *Z-*values extracted from the bilateral amygdala mask. This revealed a main effect of threat, *F*(1,20) = 13.40, *P* = .002, *η*_p_^2^ = .40, with stronger activation for threatening situations (*M* = .21, *SE* = .12) compared to neutral situations (*M* = .03, *SE* = .1), no main effect of familiarity, *F*(1,20) = .14, *P* = .711, *η*_p_^2^ = .007 and no interaction between threat and familiarity, *F*(1,20) = .24, *P* = .631, *η*_p_^2^ = .012. See Figure 3.

### Individual differences

**Fig. 4:**
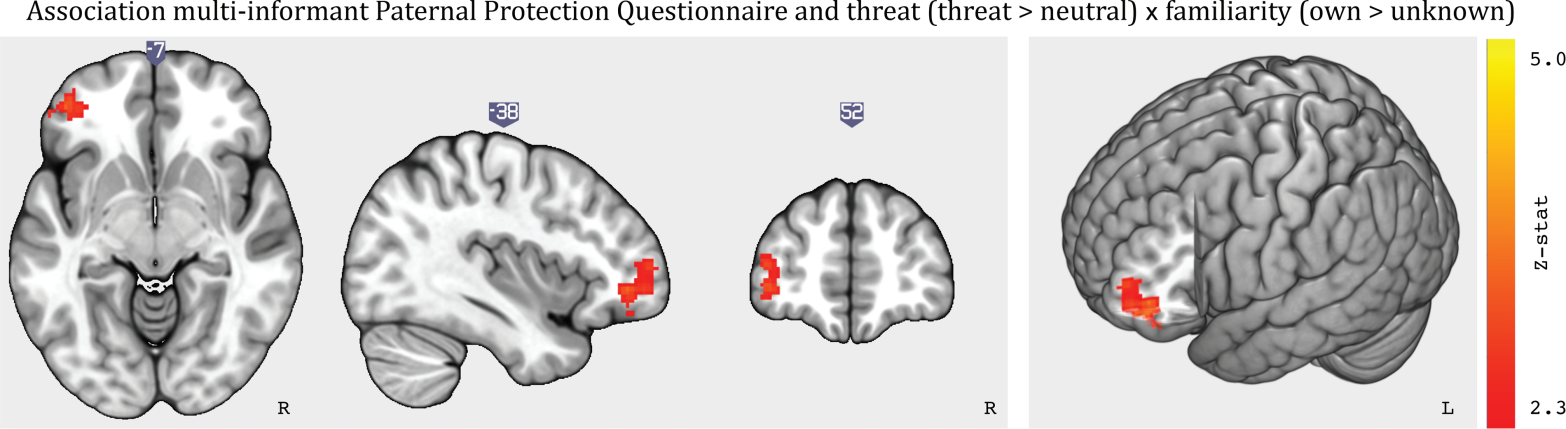
Positive association in the Frontal Pole between Paternal Protection (multi-informant PPQ) and the interaction between threat (threat > neutral) and familiarity (own > unknown).

To examine the relationship between task activation and individual differences in everyday actions of protecting the unborn child we performed a whole-brain analysis on the contrasts of threat *vs* neutral and the interaction between threat and familiarity for the pre-natal session including the multi-informant PPQ scores as a continuous predictor. We found a significant one-directional association for the contrast in which the interaction was defined as threat > neutral and own > unknown. As illustrated in Figure 4, paternal protection was associated with the interaction between threat and familiarity in one cluster within the frontal pole (*P* = .016, see Supplementary Table 3). That is, participants who were more protective in daily life showed higher frontal pole activation for their own (*vs* an unknown) infant in threatening (*vs* neutral) situations.

Additionally, as suggested by a reviewer, we examined whether the multi-informant PPQ score was associated with the activation when viewing one’s own infant in a threatening situation within the three clusters where we found activation for the interaction between threat and familiarity. We did not find a significant association (see Supplementary data).

### Longitudinal analysis

**Fig. 5:**
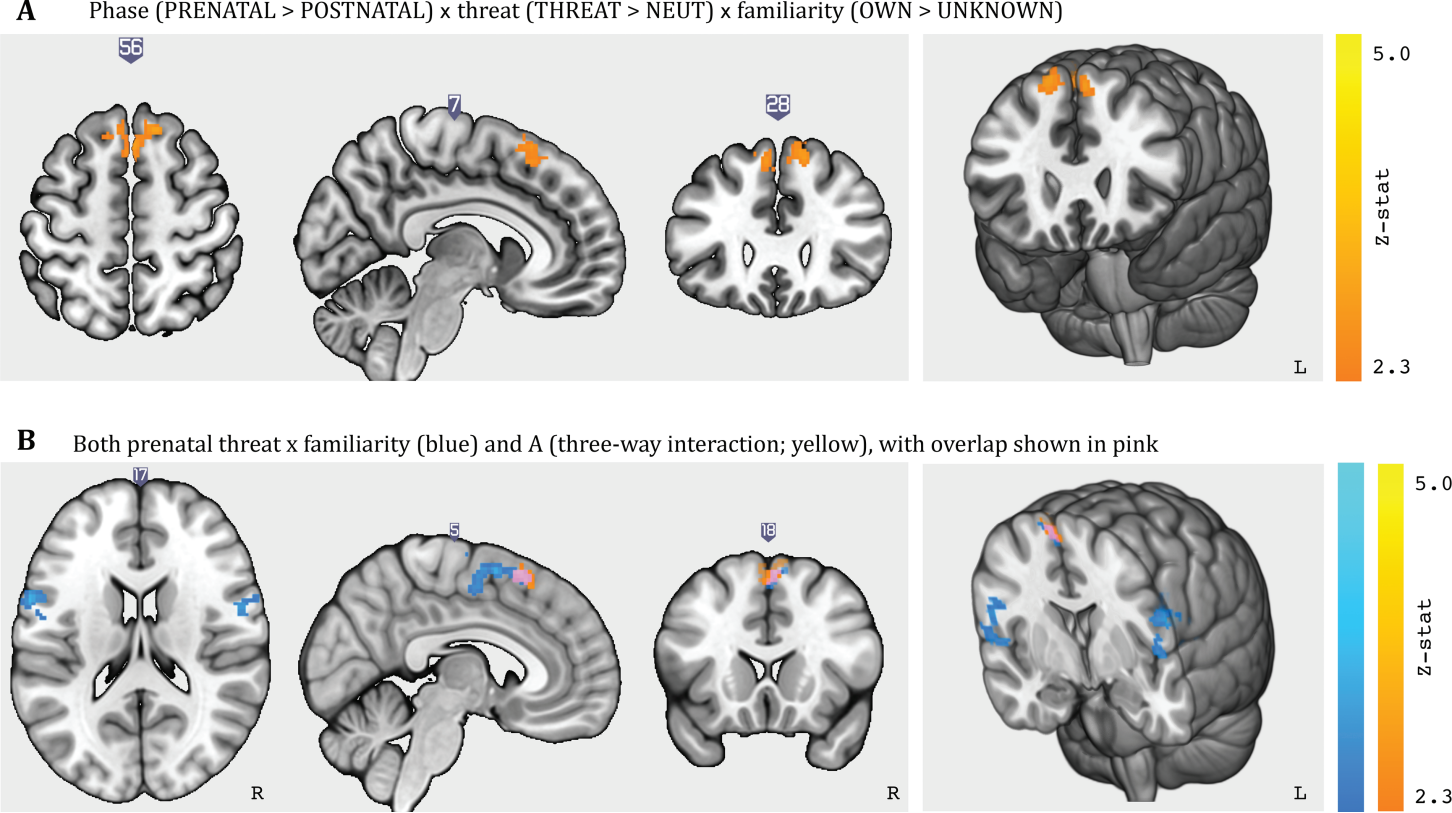
Activation for (A) three-way interaction phase × threat × familiarity, where the interaction effect between threat and familiarity was stronger in the pre-natal compared to post-natal session, and (B) both the three-way interaction and the pre-natal threat × familiarity interaction (see also Figure 2B) overlaid on standard brain. Results (cluster-thresholded at 2.3, *P* < .05) are displayed on MNI-normalized template brain (i.e. mni152_2009bet.nii) using MRIcroGL software.

Subsequently, we compared the pre-natal and post-natal sessions. There were no significant differences on the main effects; however, there was a significant difference between pre- and post-natal sessions for the interaction of threat and familiarity in one cluster comprising the SFG (*P* = .012). This contrast suggested that, in this particular cluster, the interaction between threat and familiarity was no longer present in the post-natal session (Figure 5 and Supplementary Table 4). In other words, post-natally, this part of the SFG no longer showed higher activation for participants’ own (*vs* unknown) infant in threatening (*vs* neutral) situations.

## Discussion

Parental protection contributes critically to an infant’s survival, and correspondingly parents seem equipped to be sensitive to threats to their infants. To our knowledge, the current study is the first to explore the neural processes associated with paternal protection. Large regions of the brain were activated when expectant fathers viewed videos of infant-threatening (*vs* neutral) situations. These neural mechanisms depended in part on infant familiarity, with expectant fathers showing more activation in the bilateral pre-central gyrus and the juxtapositional lobule cortex when their own—compared to an unknown—infant was endangered. Post-natal results were similar, except for one region (located in the SFG) where this modulation faded. Furthermore, threat processing was related to everyday protective behavior, suggesting that fathers-to-be who protect their unborn child more also tend to display a stronger neural response to their own infant in threatening situations. However, because this is a first and exploratory study, it should be emphasized that these findings require pre-registered high-power replication.

Our primary interest was identifying the neural systems implicated when fathers perceive an infant in need of protection. We thus specifically focused on the processing of infant-threatening situations (Hahn-Holbrook *et al*., 2011), not on detection and processing of threat to the participant himself or to another adult. In other words, the task was designed to elicit the process of perceiving an infant in need of protection for which a behavioral action might be required and imagined. We found increased activations for infant-threatening situations compared to neutral situations in a variety of cortical and subcortical areas. Next to basic visual processing areas (e.g. the lateral occipital cortex), infant-threatening situations elicited activation in areas that facilitate directing attention to the threat and reacting to it, such as the posterior cingulate in combination with frontoparietal regions, which have been implicated with sensorimotor and action control (Eickhoff *et al*., 2010; Leech *et al*., 2012; Ptak, 2012). Additionally, observing infant-threatening situations elicited activation in areas previously characterized as part of a parental care network, such as the insula, SFG and the anterior cingulate cortex, which facilitate processing emotional information and simulating affective states of others (Singer *et al*., 2009; Atzil *et al*., 2012; Lindquist *et al*., 2012; Swain *et al*., 2014; Decety, 2015). On top of this, ROI analysis indicated that infant-threatening situations, compared to neutral situations, also elicited more activation in the amygdalae, strengthening the idea that fathers allotted affective salience to these situations. The amygdalae have been consistently associated with emotion processing and salience and threat detection and may play a regulating role in attention and perception (Phelps, 2006; Sergerie *et al*., 2008; Hrybouski *et al*., 2016). It seems conceivable that a possible role of the amygdala in protective behavior is to identify potential threats, after which action can be taken to ensure child safety.

In regions partly overlapping with the abovementioned findings, the effect of threat was modulated by infant familiarity. Specifically, for one’s own infant compared to an unknown infant, threatening situations activated parts of the juxtapositional lobule cortex (formerly known as the SMA/pre-SMA) and the pre-central gyri (bilaterally), areas within the larger area of the motor cortex. These regions are involved in the preparation of motor responses (e.g. Brass and von Cramon, 2002; Cunnington *et al*., 2005). If such patterns are replicated in future studies, it may reflect more preparation to protect one’s own—compared to an unknown—infant in danger. Previous work in social neuroscience has likewise found a different neural response when processing own *vs* other infants (e.g. Bartels and Zeki, 2004; Swain *et al*., 2008; Kuo *et al*., 2012), and theoretical evolutionary work suggests both parenting and prosocial behavior are modulated by kinship (e.g. DeBruine *et al*., 2016; Decety *et al*., 2016). However, more neuroimaging studies in this domain are needed to accumulate consistent patterns.

Our results further indicate that once the participants’ children were born, the modulatory effect of familiarity did not differ except for one part of the SFG—an area previously associated with cognitive control and execution (Li *et al*., 2013)—where it was dampened. One *post hoc* explanation is that once their child is born it may take fathers less cognitive effort to follow the instructions of imagining their child in threatening situations. Alternatively, responses to one’s own infant in threatening situations may have been dampened during the post-natal visit because fathers were already familiar with the task. Future replications and extensions may reveal whether these plausible mechanisms can be reliably uncovered with methods such as those used here and whether other findings also point at, for instance, a more generalized protective response once men become fathers.

In addition to describing neural networks involved in processing threat to the infant, the current study aimed to link these networks to everyday protective behaviors. Protective behavior was associated with the neural response in reaction to imagining one’s own (*vs* unknown) infant in threatening (*vs* neutral) situations. Being able to relate neural data to behavioral data collected outside of the scanner provides additional support for the validity of a study. A compelling—albeit speculative—hypothesis is that individual differences in everyday protective behavior indeed reflect that some fathers-to-be are already more attuned to threats to their child and that this is related to their neural response to infant-threatening stimuli. We found this association in a part of the frontal pole, a region that is currently poorly understood in terms of its function. There are indications that this region is involved in the coordination of multiple tasks (Kovach *et al*., 2012; Pollman *et al*., 2007). It should be noted that this association was found with the activation map for the contrast that modeled the interaction of threat > neutral and own > unknown (whole-brain level) and not the omnibus test for all contrasts, and future studies need to investigate it with more statistical power.

To our knowledge, this is the first examination of neural processes involved in parental protective behavior. Strengths of this study include the longitudinal, multi-method assessment (neuroimaging, as well as multi-informant self- and other reports) of paternal protection including a newly developed task—designed to elicit neural responses to infants in danger—that used non-static stimuli (professionally developed videos). However, several aspects of this study could be improved in future research. First, our sample size was relatively small and given that our analyses were exploratory, the results require replication. We also used a statistical threshold of *Z* > 2.3 with a cluster corrected threshold of *P* < .05, and recent studies have shown that for these thresholds the false positive rate is often above the desired level (Woo *et al*., 2014; Eklund *et al*., 2016). Although FSL’s Flame 1—the method we used here—produced conservative results in non-task data (Eklund *et al*., 2016), for task data the false positive rate is likely inflated. Therefore, the liberal threshold that was used in the current exploratory study may have increased the probability of false positives, and replications as well as more (simulation) studies on the robustness of different data analysis methods are necessary. Furthermore, for obvious ethical reasons we had to use a doll to record the videos showing threat to an infant. Care was taken to make the videos as realistic as possible but seeing real infants in real danger may elicit stronger or different responses.

Moreover, our participants were instructed to imagine that the infant depicted in the videos was either their own or someone else’s, and the neural response we measured depends on their ability to do this. Even though the in-scanner self-reports suggest that participants were successful at imagining the infant in the videos (their ratings were above the midpoint of the scale, see Supplementary data), this self-report may not accurately reflect participants’ imagination skills, and the task may be measuring ability to imagine own *vs* unknown infant in addition to or rather than a differential response to threat to these infants. We did, however, support the participants’ imagination by showing a morphed picture of what their future child might look like. This was based on previous indications that facial resemblance, as a cue for kinship, is associated with willingness to invest in a child (e.g. DeBruine, 2004; Platek *et al*., 2004). Given the topic (reactions to infant-threatening situations) and the pre-natal and early post-natal period of interest, this seemed the most valid way to design our task. However, the fact remains that fathers did not truly see their own infant in danger. Future studies could complement our findings by using an actual picture of the father’s child (post-natally) or to measure the ability to imagine their child in different ways, in order to establish how this is related to being protective (e.g. are more protective fathers better at imagining their own, *vs* an unknown, infant in danger?).

In a similar vein, although the PPQ that we used to measure protective behaviors pre-natally is meant to measure protection of the child, the items mostly concern behaviors directed at the mother because she is carrying the child. This means that this questionnaire could be picking up social attunement to the partner rather than paternal protection. The development and validation of the PPQ is still ongoing, and a recurrent question remains how these aspects can be untied and whether they should be. One possibility is that pre-natal social attunement to the partner is one of the many aspects of paternal protection, or that it serves as a precursor of future paternal protection. Both behavioral and brain studies are needed to answer these questions in the relatively uncharted domain of fatherhood.

The aim of the current study was to take a first step in mapping paternal protection. We did so by employing a multi-method approach focussing on the neural basis for the preparatory phase of this behavior. In a broader sense, the inclination to protect children can be seen as a fundamental form of prosocial behavior, yet as such it has received little attention so far. Future work might productively focus on replicating the current study in a larger independent sample and could examine protection in fathers, mothers and non-parents, to find possible similarities and differences in the underlying processes. Of particular interest may be to examine how individual differences in protective behavior relate to personality and situational factors, and whether there is an underlying continuum with insensitive parenting and neglect (van IJzendoorn and Bakermans-Kranenburg, 2018). Future investigations may try to uncover how protective behavior as measured by our PPQ relates to overprotective parenting, which is assumed to influence child development negatively (Ungar, 2009). By furthering knowledge on the psychological and neurobiological dimensions of paternity, this type of work may have important implications for developmental, behavioral and societal aspects of parental care.

## Supplementary Material

scan-18-286-File007_nsz018.docx
