# Supplementary material for: Exploring the neural basis for paternal protection: an investigation of the neural response to infants in danger
Source: Soc Cogn Affect Neurosci. 2019 Mar 7;14(4):447–57. doi: 10.1093/scan/nsz018 (PMC6523437; doi:10.1093/scan/nsz018)

**Index of supplementary material:**

In-scanner ratings, prenatal session, pages 2 – 5

In-scanner ratings, postnatal session, pages 5 – 8

Supplementary Table 1, page 9

Supplementary Table 2, page 10

Supplementary Table 3, page 11

Supplementary Table 4, page 11

Additional details about morphed pictures, page 12

Additional analyses with scan order in the model upon reviewer request, page 13

Additional analyses correlation PPQ and mean Z functional ROIs upon reviewer request, page 13

Visual comparison of prenatal and postnatal findings, page 14

**Supplementary material – In-scanner ratings**

Prenatal session

*Supplementary Figure 1. In-scanner ratings of arousal (left) and valence (right) as a function of threat (threat vs. neutral) by familiarity (own vs. unknown infant).*

**Arousal.** A 2(threat: threat vs. neutral) × 2(familiarity: own vs. unknown infant) × 2(time point in the task: beginning vs. end) repeated measures ANOVA on participants’ arousal ratings in the prenatal session revealed there was a main effect of threat, *F*(1,20) = 54.62, *p* < .001, *η*_p_^2^ = .73, and a marginally significant main effect of familiarity, *F*(1,20) = 4.26, *p* = .052, *η*_p_^2^ = .18, which were qualified by an interaction between threat and familiarity, *F*(1,20) = 9.98, *p* = .005, *η*_p_^2^ = .33. There was no main effect of time point, *F*(1,20) = .46, *p* = .51, *η*_p_^2^ = .02, no interaction between time point and threat, *F*(1,20) = 2.53, *p* = .13, *η*_p_^2^ = .11, no interaction between time point and familiarity, *F*(1,20) = 2.62, *p* = .12, *η*_p_^2^ = .12, and no interaction between time point, threat and familiarity, *F*(1,20) = 2.77, *p* = .11, *η*_p_^2^ = .12.

Subsequent pairwise comparisons (Bonferroni corrected) indicated that for threatening situations, participants felt more tense when imagining their own infant (*M* = 69.21, *SE* = 3.98) compared to an unknown infant (*M* = 55.38, *SE* = 4.74), *F*(1,20) = 13.35, *p* = .002, *η*_p_^2^ = .40, 95% CI [5.94, 21.73], while for neutral situations, there was no difference between own (*M* = 25.29, *SE* = 4.07) and unknown infant (*M* = 28.17, *SE* = 4.05), *F*(1,20) = .60, *p* = .446, *η*_p_^2^ = .03, 95% CI [-10.61, 4.85]. For both their own and the unknown infant, participants felt significantly more tense after watching threatening situations compared to neutral situations, *F*(1,20) = 50.92, *p* < .0001, *η*_p_^2^ = .72, 95% CI [ 31.09, 56.77], and *F*(1,20) = 33.06, *p* < .0001, *η*_p_^2^ = .62, 95% CI [ 17.33, 37.09], respectively. See Supplementary Figure 1.

**Valence.** A 2(threat: threat vs. neutral) × 2(familiarity: own vs. unknown infant) × 2(time point in the task: beginning vs. end) repeated measures ANOVA on participants’ valence ratings in the prenatal session revealed a main effect of threat, *F*(1,20) = 79.04, *p* < .0001, *η*_p_^2^ = .80, no main effect of familiarity, *F*(1,20) = .005, *p* = .95, *η*_p_^2^ < .001, no main effect of time point, *F*(1,20) = .59, *p* = .45, *η*_p_^2^ = .03, an interaction between threat and familiarity, *F*(1,20) = 23.36, *p* = .0001, *η*_p_^2^ = .54, an interaction between threat and time point, *F*(1,20) = 16.42, *p* = .001, *η*_p_^2^ = .45, and no interaction between time point and familiarity or time point, threat and familiarity, *F*(1,20) = 1.95, *p* = .18, *η*_p_^2^ = .09 and *F*(1,20) = .65, *p* = .43, *η*_p_^2^ = .03, respectively. Subsequent pairwise comparisons (Bonferroni corrected) of the threat × familiarity interaction indicated that for threatening situations, participants felt more negative when imagining their own infant (*M* = 19.02, *SE* = 3.71) compared to the unknown infant (*M* = 30.12, *SE* = 3.79), *F*(1,20) = 26.21, *p* < .0001, *η*_p_^2^ = .57, 95% CI [ -15.62, -6.57], and for neutral situations, participants felt more positive when imagining their own infant (*M* = 74.62, *SE* = 3.33) compared to the unknown infant (*M* = 63.81, *SE* = 3.02), *F*(1,20) = 8.32, *p* = .009, *η*_p_^2^ = .29, 95% CI [2.99, 18.63]. For both their own and unknown infant, participants felt significantly more negative after watching threatening situations compared to neutral situations, *F*(1,20) = 88.16, *p* < .0001, *η*_p_^2^ = .82, 95% CI [-67.95, -43.24] and *F*(1,20) = 44.23, *p* < .0001, *η*_p_^2^ = .69, 95% CI [-44.25, -23.19], respectively. See Supplementary Figure 1.

Subsequent pairwise comparisons (Bonferroni corrected) of the threat × time point interaction indicated that for threatening situations, participants felt more negative at the beginning (*M* = 20.60, *SE* = 3.96) compared with the end (*M* = 28.55, *SE* = 3.57) of the task, *F*(1,20) = 11.87, *p* = .003, *η*_p_^2^ = .37, 95% CI [ -12.77, -3.14], while for neutral situations, there was no significant difference between the beginning (*M* = 71.69, *SE* = 2.98) and the end (*M* = 66.74, *SE* = 2.83) of the task, *F*(1,20) = 3.34, *p* = .083, *η*_p_^2^ = .14, 95% CI [ -.72, 10.62]. Importantly, in both the beginning and the end of the task, participants felt significantly more negative after watching threatening situations compared to neutral situations, *F*(1,20) = 96.22, *p* < .0001, *η*_p_^2^ = .83, 95% CI [ -61.69, -40.23] and *F*(1,20) = 51.41, *p* < .0001, *η*_p_^2^ = .72, 95% CI [ -49.30, -27.08], respectively.

**Imagining own vs. unknown infant.** A 2(threat: threat vs. neutral) × 2(familiarity: own vs. unknown infant) × 2(time point in the task: beginning vs. end) repeated measures ANOVA on participants’ ratings of how well they could imagine their own or someone else’s infant in the videos in the prenatal session revealed no main effect of threat, *F*(1,20) = 1.41, *p* = .25, *η*_p_^2^ = .07, no main effect of familiarity, *F*(1,20) = .61, *p* = .44, *η*_p_^2^ = .03, no main effect of time point in task, *F*(1,20) = 4.28, *p* = .052, *η*_p_^2^ = .18, no interaction between threat and familiarity, *F*(1,20) = .036, *p* = .851, *η*_p_^2^ = .002, no interaction between threat and round, *F*(1,20) = .12, *p* = .73, *η*_p_^2^ = .006, an interaction between familiarity and time point, *F*(1,20) = 11.62, *p* = .003, *η*_p_^2^ = .38, and no interaction between threat, familiarity and time point, *F*(1,20) = 1.41, *p* = .25, *η*_p_^2^ = .07.

Subsequent pairwise comparisons (Bonferroni corrected) of the familiarity × time point interaction indicated that at the beginning of the task, participants reported to do worse at imagining their own infant (*M* = 64.36, *SE* = 4.40) compared to the unknown infant (*M* = 72.43, *SE* = 3.41), *F*(1,20) = 5.08, *p* = .036, *η*_p_^2^ = .20, 95% CI [ -15.55, -.60], while at the end of the task, there was no difference between imagining own (*M* = 65.43, *SE* = 3.72) or unknown (*M* = 62.95, *SE* = 3.68) infant, *F*(1,20) = .35, *p* = .56, *η*_p_^2^ = .02. Participants did not do better at imagining their own infant in the beginning compared the end of the task, *F*(1,20) = .24, *p* = .63, *η*_p_^2^ = .012, 95% CI [ -5.68, 3.54], while they did report to do better at imagining the unknown infant at the beginning compared to the end of the task, *F*(1,20) = 10.99, *p* = .003, *η*_p_^2^ = .36, 95% CI [ 3.51, 15.44].

These results are highly comparable to the response data of the postnatal session (see below).

Postnatal session

**Arousal.** A 2(threat: threat vs. neutral) × 2(familiarity: own vs. unknown infant) × 2(time point in the task: beginning vs. end) repeated measures ANOVA on participants’ arousal ratings in the postnatal session revealed there was a main effect of threat, *F*(1,16) = 47.27, *p* < .0001, *η*_p_^2^ = .75, and a marginally significant main effect of familiarity, *F*(1,16) = 3.82, *p* = .068, *η*_p_^2^ = .19, which were qualified by an interaction between threat and familiarity, *F*(1,16) = 13.51, *p* = .002, *η*_p_^2^ = .46. The main effect of time point was not significant, *F*(1,16) =2.78, *p* = .115, *η*_p_^2^ = 15, there was an interaction between time point and familiarity, *F*(1,16) =5.27, *p* = .036, *η*_p_^2^ = .25, and no interaction between time point, threat and familiarity, *F*(1,16) = .17, *p* = .68, *η*_p_^2^ = .01. Subsequent pairwise comparisons (Bonferroni corrected) of the threat × familiarity interaction indicated that for threatening situations, participants felt more tense when imagining their own infant (*M* = 67.77, *SE* = 4.73) compared to the unknown infant (*M* = 57.24, *SE* = 4.25), *F*(1,16) = 15.14, *p* = .001, *η*_p_^2^ = .49, 95% CI [4.79, 16.28], while for neutral situations, there was no difference between own (*M* = 27.38, *SE* = 4.92) and unknown infant (*M* = 30.74, *SE* = 4.51), *F*(1,16) = 1.72, *p* = .209, *η*_p_^2^ = .097, 95% CI [-8.78, 2.07]. ]. For both their own and the unknown infant, participants felt significantly more tense after watching threatening situations compared to neutral situations, *F*(1,16) = 47.31, *p* < .0001, *η*_p_^2^ = .75, 95% CI [ 27.94, 52.83], and *F*(1,16) = 35.15, *p* < .0001, *η*_p_^2^ = .69, 95% CI [ 17.02, 35.98], respectively.

Subsequent pairwise comparisons (Bonferroni corrected) of the familiarity × time point interaction indicated that at the beginning of the task, participants felt more tense when imagining their own infant (*M* = 47.44, *SE* = 3.78) compared to the unknown infant (*M* =38.74, *SE* = 4.08), *F*(1,16) = 8.63, *p* = .010, *η*_p_^2^ = .35, 95% CI [ 2.42, 14.99], while at the end of the task, there was no difference between own (*M* = 47.71, *SE* = 4.45) and other infant (*M* = 49.24, *SE* = 4.74), *F*(1,16) = .30, *p* = .594, *η*_p_^2^ = .02, 95% CI [ 2.42, 14.99].

**Valence.** A 2(threat: threat vs. neutral) × 2(familiarity: own vs. unknown infant) × 2(time point in the task: beginning vs. end) repeated measures ANOVA on participants’ valence ratings in the postnatal session revealed a main effect of threat, *F*(1,16) = 53.48, *p* < .001, *η*_p_^2^ = .77, no main effect of familiarity, *F*(1,16) = 1.85, *p* = .19, *η*_p_^2^ = .10, no main effect of time point, *F*(1,16) = 2.23, *p* = .155, *η*_p_^2^ = .12, an interaction between threat and familiarity, *F*(1,16) = 37.00, *p* < .001, *η*_p_^2^ = .70, an interaction between threat and time point, *F*(1,16) = 6.51, *p* = .021, *η*_p_^2^ = .30, and no interaction between time point and familiarity or time point, threat and familiarity, *F*(1,16) = .02, *p* = .90, *η*_p_^2^ = .001 and *F*(1,16) = .51, *p* = .49, *η*_p_^2^ = .03, respectively. Subsequent pairwise comparisons (Bonferroni corrected) of the threat × familiarity interaction indicated that for threatening situations, participants felt more negative when imagining their own infant (*M* = 20.53, *SE* = 4.89) compared to the unknown infant (*M* = 27.71, *SE* = 4.05), *F*(1,16) = 8.55, *p* < .010, *η*_p_^2^ = .59, 95% CI [ -12.38, -1.97], and for neutral situations, participants felt more positive when imagining their own infant (*M* = 78.44, *SE* = 2.45) compared to the unknown infant (*M* = 66.03, *SE* = 4.29), *F*(1,16) = 23.44, *p* < .001, *η*_p_^2^ = .59, 95% CI [6.98, 17.85]. For both their own and the unknown infant, participants felt significantly more negative after watching threatening situations compared to neutral situations, *F*(1,16) = 75.50, *p* < .0001, *η*_p_^2^ = .83, 95% CI [-72.04, -43.78] and *F*(1,16) = 31.01, *p* < .0001, *η*_p_^2^ = .66, 95% CI [-52.91, -23.74], respectively.

Subsequent pairwise comparisons (Bonferroni corrected) of the threat × time point interaction indicated that for threatening situations, participants did not feel more negative at the beginning (*M* = 22.65, *SE* = 4.10) compared with the end (*M* = 25.59, *SE* = 4.70) of the task, *F*(1,16) = 2.75, *p* = .12, *η*_p_^2^ = .15, 95% CI [ -6.70, .82], while for neutral situations, participants did feel more positive at the beginning (*M* = 76.47, *SE* = 3.20) compared to the end (*M* = 68.00, *SE* = 4.21) of the task, *F*(1,16) = 5.23, *p* = .036, *η*_p_^2^ = .25, 95% CI [.62, 16.32]. Importantly, in both the beginning and the end of the task, participants felt significantly more negative after watching threatening situations compared to neutral situations, *F*(1,16) = 70.66, *p* < .0001, *η*_p_^2^ = .82, 95% CI [ -67.40, -40.25] and *F*(1,20) = 32.36, *p* < .0001, *η*_p_^2^ = .67, 95% CI [ -58.22, -26.61], respectively.

**Imagining own vs. someone else’s infant.** A 2(threat: threat vs. neutral) × 2(familiarity: own vs. unknown infant) × 2(time point in the task: beginning vs. end) repeated measures ANOVA on participants’ ratings of how well they could imagine their own or someone else’s infant in the videos during the postnatal session revealed no main effect of threat, *F*(1,16) = 1.58, *p* = .23, *η*_p_^2^ = .09, no main effect of familiarity, *F*(1,16) = .68, *p* = .42, *η*_p_^2^ = .04, no main effect of time point in task, *F*(1,16) = 1.14, *p* = .30, *η*_p_^2^ = .07, no interaction between threat and familiarity, *F*(1,16) = .16, *p* = .69, *η*_p_^2^ = .01, no interaction between threat and time point, *F*(1,16) = .10, *p* = .76, *η*_p_^2^ = .006, no interaction between familiarity and time point, *F*(1,16) = 2.84, *p* = .11, *η*_p_^2^ = .15, and no interaction between threat, familiarity and time point, *F*(1,16) = .03, *p* = .87, *η*_p_^2^ = .002.

Supplementary Table 1

*Local maxima for the main effect of threat (threat vs. neutral)*

|  |  |  | MNI coordinates | | |  |  |  |
| --- | --- | --- | --- | --- | --- | --- | --- | --- |
| Cluster | Region | Size | x | y | z | *z* | *p* |  |
| 1 | L parietal operculum cortex | 58966 | -56 | -36 | 26 | 6.55 | 0 |  |
|  | L posterior cingulate gyrus |  | -12 | -30 | 38 | 6.46 |  |  |
|  | R lingual gyrus |  | 6 | -76 | -8 | 6.03 |  |  |
|  | L occipital pole |  | -10 | -100 | 12 | 5.94 |  |  |
|  | R lateral occipital cortex |  | 46 | -74 | 4 | 5.91 |  |  |
|  | R juxtapositional lobule cortex/WM |  | 10 | 2 | 62 | 5.88 |  |  |
| 2 | L MFG | 532 | -38 | 32 | 22 | 4.66 | .003 |  |
|  | L MFG |  | -40 | 36 | 26 | 4.16 |  |  |
|  | L MFG |  | -40 | 30 | 34 | 3.92 |  |  |
|  | L frontal pole/MFG |  | -38 | 38 | 30 | 3.84 |  |  |
|  | L WM/MFG |  | -34 | 34 | 20 | 3.75 |  |  |
|  | L WM |  | -26 | 28 | 26 | 2.7 |  |  |
| 3 | R WM/lateral ventricle | 337 | 22 | -44 | 12 | 4.33 | .046 |  |
|  | R WM/lateral ventricle |  | 14 | -34 | 20 | 3.83 |  |  |
|  | R WM/lateral ventricle |  | 32 | -48 | 8 | 3.73 |  |  |
|  | R WM/lateral ventricle |  | 18 | -38 | 18 | 3.58 |  |  |
|  | R WM/lateral ventricle |  | 28 | -38 | 6 | 2.64 |  |  |
|  | R WM/lateral ventricle |  | 14 | -24 | 24 | 2.51 |  |  |

Note. Table displays the local maxima per cluster and is not a conclusive list of the significant regions. MFG = middle frontal gyrus; WM = white matter.

Supplementary Table 2

*Local maxima for the interaction of threat (threat vs. neutral) × familiarity (own vs. unknown infant)*

|  |  |  | MNI coordinates | | |  |  |  |
| --- | --- | --- | --- | --- | --- | --- | --- | --- |
| Cluster | Region | Size | x | y | z | *z* | *p* |  |
| 1 | L precentral gyrus | 438 | -60 | 6 | 16 | 3.83 | .006 |  |
|  | L central opercular cortex |  | -56 | -2 | 6 | 3.42 |  |  |
|  | L precentral gyrus |  | -54 | 0 | 22 | 3.01 |  |  |
|  | L temporal pole |  | -58 | 8 | -4 | 2.98 |  |  |
|  | L postcentral gyrus |  | -58 | -6 | 16 | 2.94 |  |  |
|  | L central opercular gyrus |  | -50 | 2 | 0 | 2.93 |  |  |
| 2 | R juxtapositional lobule cortex | 407 | 0 | 0 | 60 | 3.57 | .010 |  |
|  | R juxtapositional lobule cortex |  | 6 | 8 | 60 | 3.34 |  |  |
|  | R SFG |  | 4 | 18 | 60 | 3.19 |  |  |
|  | L juxtapositional lobule cortex |  | -6 | -4 | 64 | 3.17 |  |  |
|  | L juxtapositional lobule cortex |  | -8 | 2 | 58 | 3.12 |  |  |
|  | R SFG |  | 2 | 22 | 56 | 3.03 |  |  |
| 3 | R WM/precentral gyrus | 319 | 54 | 2 | 18 | 3.03 | .041 |  |
|  | R central opercular cortex |  | 60 | 0 | 6 | 3.01 |  |  |
|  | R precentral gyrus |  | 54 | 6 | 10 | 2.96 |  |  |
|  | R precentral gyrus |  | 54 | 6 | 24 | 2.94 |  |  |
|  | R central opercular cortex |  | 50 | -6 | 6 | 2.89 |  |  |
|  | R precentral gyrus |  | 62 | 8 | 22 | 2.87 |  |  |

Note. Table displays the local maxima per cluster and is not a conclusive list of the significant regions. SFG = superior frontal gyrus; WM = white matter.

Supplementary Table 3

*Local maxima for the association between the multi-informant Paternal Protection Questionnaire (PPQ) scores and the interaction between threat (threat vs. neutral) and familiarity (own vs. unknown infant)*

|  |  |  | MNI coordinates | | |  |  |  |
| --- | --- | --- | --- | --- | --- | --- | --- | --- |
| Cluster | Region | Size | x | y | z | *z* | *p* |  |
| 1 | L WM/frontal pole | 370 | -38 | 44 | -4 | 3.41 | .0163 |  |
|  | L frontal pole |  | -44 | 46 | -8 | 3.36 |  |  |
|  | L frontal pole |  | -44 | 50 | 8 | 3.29 |  |  |
|  | L frontal pole |  | -44 | 48 | 12 | 3.26 |  |  |
|  | L frontal pole |  | -42 | 50 | -4 | 3.20 |  |  |
|  | L frontal pole |  | -34 | 56 | 6 | 3.07 |  |  |

Note. Table displays the local maxima per cluster and is not a conclusive list of the significant regions. WM = white matter.

Supplementary Table 4

*Local maxima for the interaction of phase (prenatal vs. postnatal) × threat (threat vs. neutral) × familiarity (own vs. unknown infant)*

|  |  |  | MNI coordinates | | |  |  |  |
| --- | --- | --- | --- | --- | --- | --- | --- | --- |
| Cluster | Region | Size | x | y | z | *z* | *p* |  |
| 1 | R SFG | 290 | 12 | 30 | 58 | 3.07 | .0123 |  |
|  | L SFG |  | -6 | 30 | 52 | 3 |  |  |
|  | R SFG |  | 2 | 22 | 56 | 2.93 |  |  |
|  | R SFG |  | 4 | 20 | 60 | 2.93 |  |  |
|  | R SFG |  | 6 | 24 | 60 | 2.83 |  |  |
|  | L SFG |  | -6 | 38 | 48 | 2.74 |  |  |

Note. Table displays the local maxima per cluster and is not a conclusive list of the significant regions. SFG = superior frontal gyrus.

Additional details about morphed pictures

To ensure the morphed face was realistic, participants provided a photograph of themselves looking straight into the camera, with a neutral expression, and without piercings or glasses. Before morphing, photographs were edited to remove external features (i.e., beards, hair covering the face and ears). All images were resized to 640x480 pixels.

Example picture:


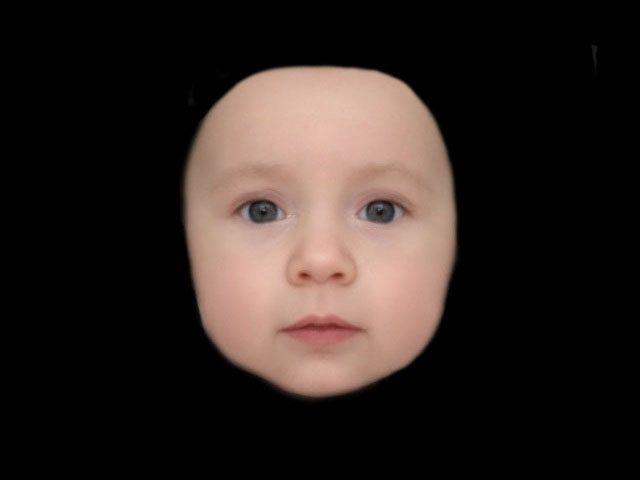


**Additional analyses with scan order in the model upon reviewer request**

An interesting suggestion was made by one of the reviewers to add scan order (whether the prenatal placebo session was the first or second time the father-to-be completed the task) in our model to see if effects differ as a function of this variable.

The group level whole-brain analysis of the prenatal session was repeated, this time with demeaned session order as an indicator value entered as an EV. Session order did not have a significant effect.

**Additional analyses correlation PPQ and mean Z functional ROIs upon reviewer request**

An interesting suggestion was made by one of the reviewers to see whether fathers with higher scores on the paternal protective score [the multi-informant PPQ] would show stronger activation in the functional ROIs of the Threat x Familiarity interaction contrast when viewing their own infant in danger.

In order to explore this suggestion, we extracted the mean Z scores of the three functional ROIs with Featquery (FSL, <https://fsl.fmrib.ox.ac.uk/fsl>) for viewing own infant in danger. The multi-informant PPQ score did not correlate significantly with the first, second or third cluster, *r*(20) = -.08, *p* = .73, *r*(20) = -.21, *p* = .37, and *r*(20) = -.15, *p* = .53, respectively (not adjusted for multiple testing).

**Visual comparison of prenatal and postnatal findings**

Below we present images of both the prenatal and postnatal session separately. These images are for visual comparison (note that these images are the result of two separate analyses, one for the prenatal session and one for the postnatal session, and therefor comparing these images does not take the within-subject nature of our study design into account).

Prenatal main effect of threat: THREAT > NEUTRAL


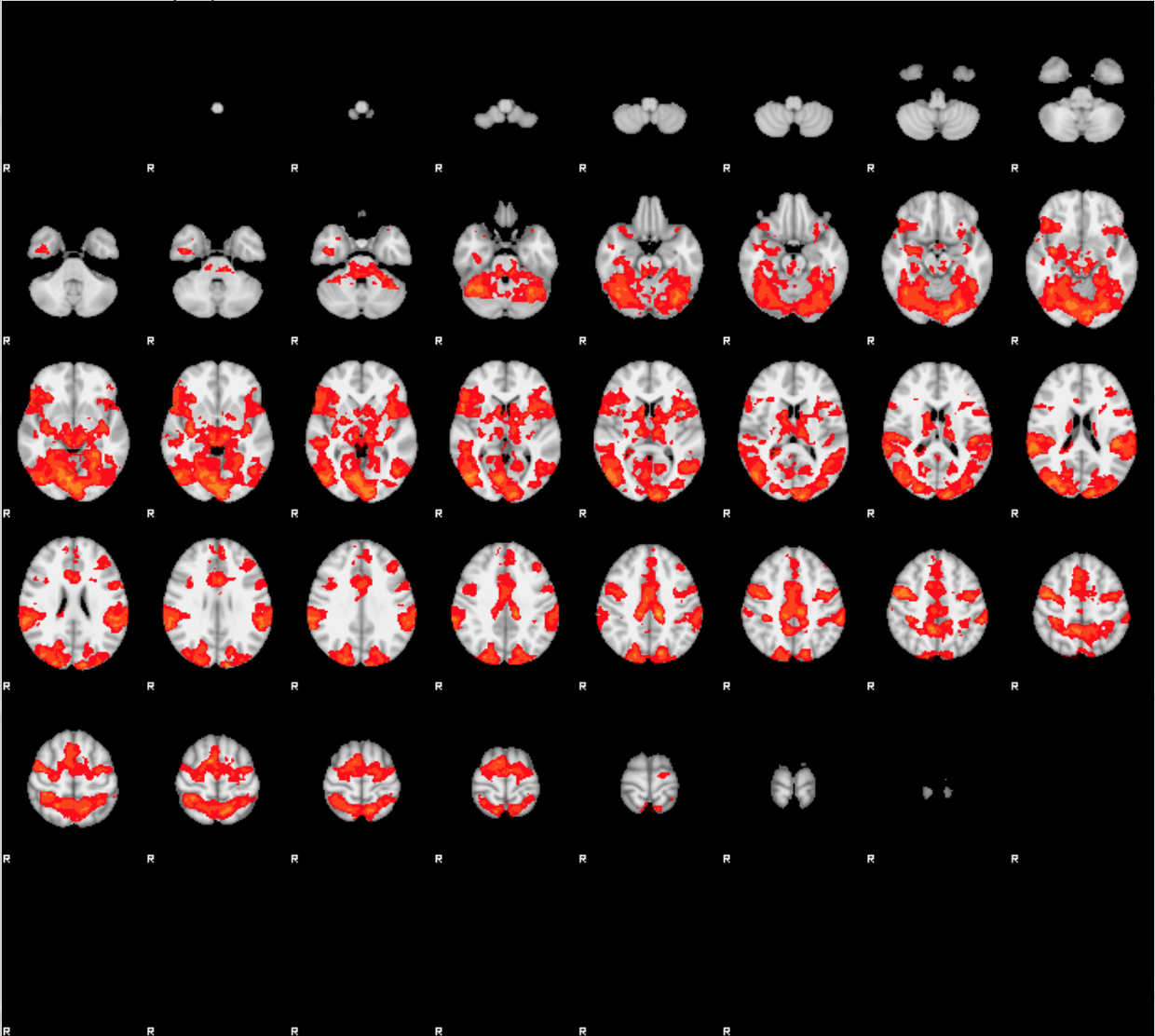


Postnatal main effect of threat: THREAT > NEUTRAL


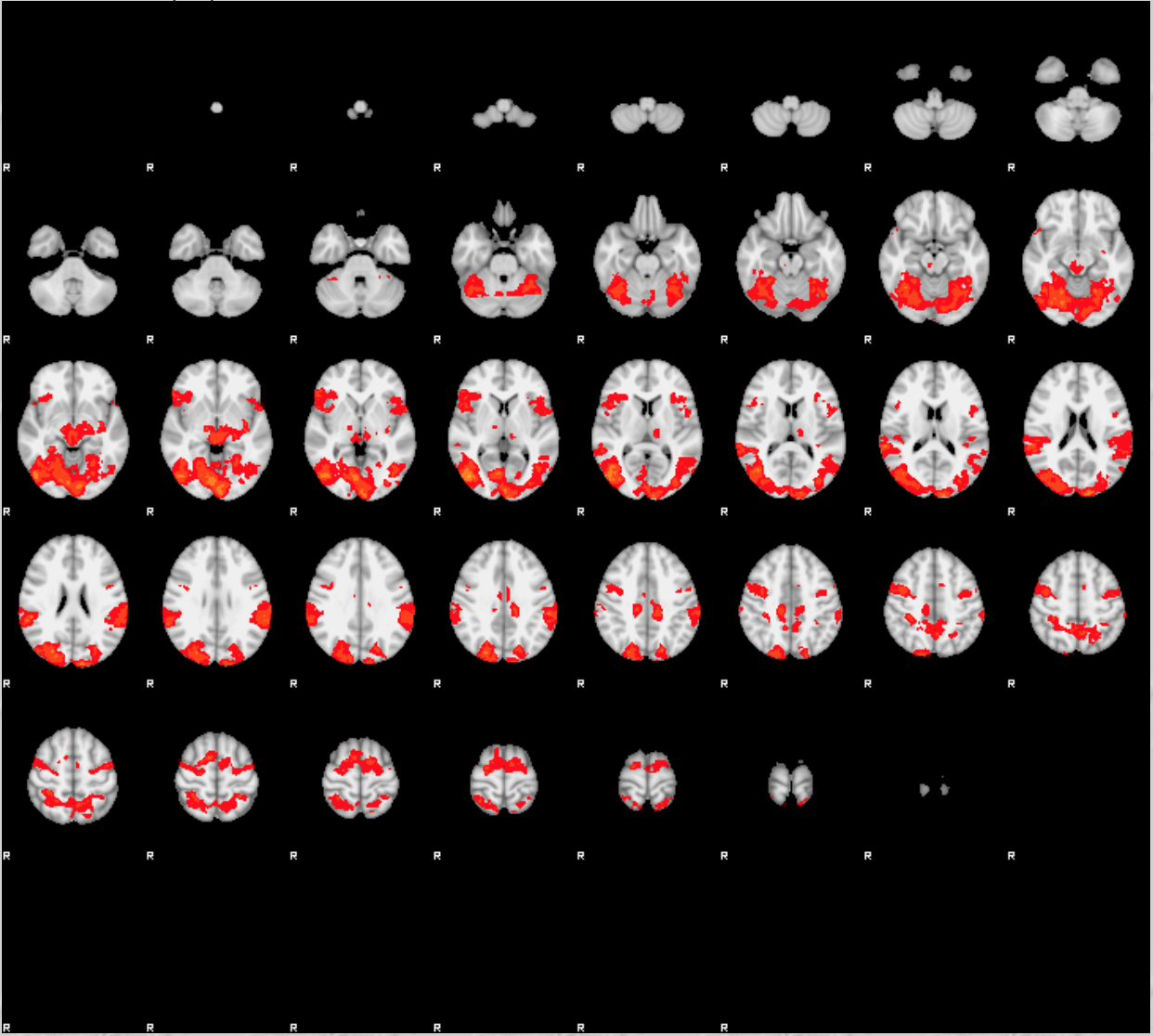


Prenatal interaction effect: THREAT > NEUTRAL x OWN > UNKNOWN


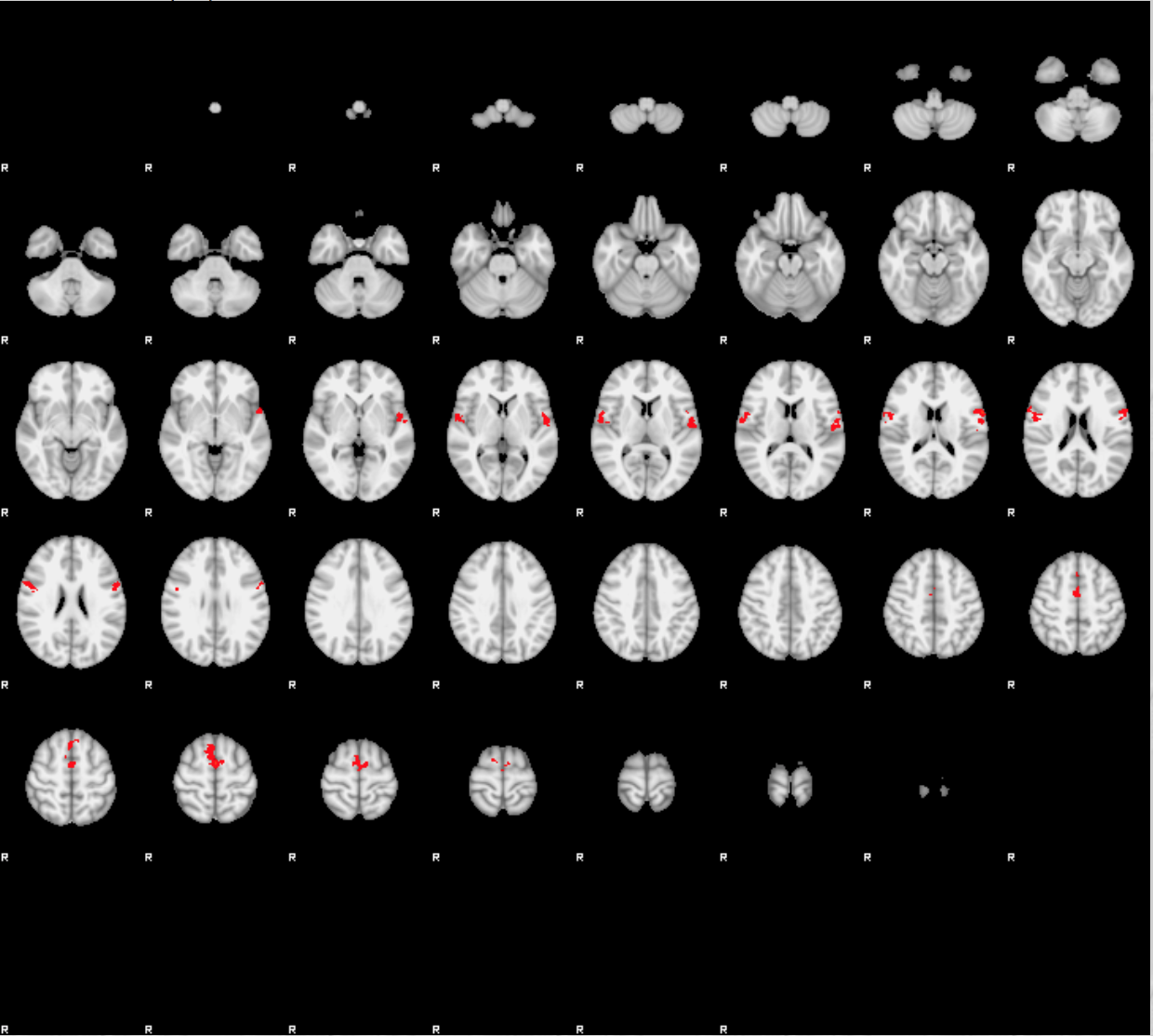


Postnatal interaction effect: THREAT > NEUTRAL x OWN > UNKNOWN
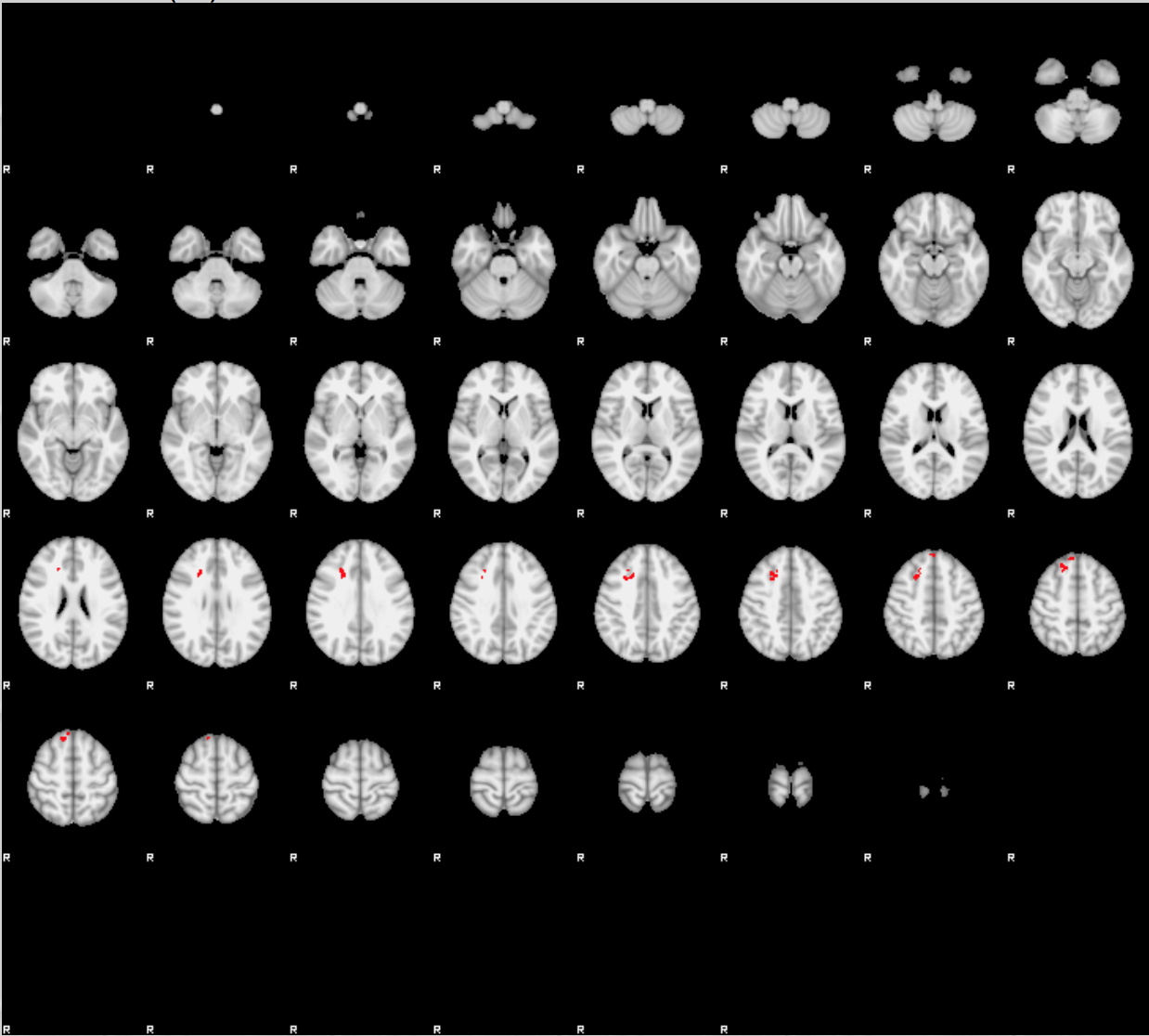

Supplement: scan-18-286-File007_nsz018.docx [file scan-18-286-file007_nsz018.docx]
